# Classifying and fact-checking health-related information about COVID-19 on Twitter/X using machine learning and deep learning models

**DOI:** 10.1186/s12911-025-02895-y

**Published:** 2025-02-11

**Authors:** Elham Sharifpoor, Maryam Okhovati, Mostafa Ghazizadeh-Ahsaee, Mina Avaz Beigi

**Affiliations:** 1 Medical Library and Information Sciences Department, Medical Informatics Research Center, Institute for Futures Studies in Health, Kerman University of Medical Sciences, Kerman, Iran; 2 Department of Computer Engineering, Shahid Bahonar University of Kerman, Kerman, Iran

**Keywords:** COVID-19, Convolutional neural networks, Deep learning, Health information management, Information dissemination, Misinformation, Machine learning, Trustworthy information

## Abstract

**Background:**

Despite recent progress in misinformation detection methods, further investigation is required to develop more robust fact-checking models with particular consideration for the unique challenges of health information sharing. This study aimed to identify the most effective approach for detecting and classifying reliable information versus misinformation health content shared on Twitter/X related to COVID-19.

**Methods:**

We have used 7 different machine learning/deep learning models. Tweets were collected, processed, labeled, and analyzed using relevant keywords and hashtags, then classified into two distinct datasets: “Trustworthy information” versus “Misinformation”, through a labeling process. The cosine similarity metric was employed to address oversampling the minority of the Trustworthy information class, ensuring a more balanced representation of both classes for training and testing purposes. Finally, the performance of the various fact-checking models was analyzed and compared using accuracy, precision, recall, and F1-score ROC curve, and AUC.

**Results:**

For measures of accuracy, precision, F1 score, and recall, the average values of TextConvoNet were found to be 90.28, 90.28, 90.29, and 0.9030, respectively. ROC AUC was 0.901.“Trustworthy information” class achieved an accuracy of 85%, precision of 93%, recall of 86%, and F1 score of 89%. These values were higher than other models. Moreover, its performance in the misinformation category was even more impressive, with an accuracy of 94%, precision of 88%, recall of 94%, and F1 score of 91%.

**Conclusion:**

This study showed that TextConvoNet was the most effective in detecting and classifying trustworthy information V.S misinformation related to health issues that have been shared on Twitter/X.

## Background

The dissemination of information has increasingly occurred through digital media [1]. In recent years, the use of social media platforms has become a significant source for gathering and studying health information. On these digital platforms, users actively share their information, opinions, and experiences regarding diseases, treatments, and other health-related topics [2]. Twitter/X is one of the most popular social media that has shared a large amount of health-related information [3]. This social media is a valuable tool for real-time monitoring of public health, including early detection and intervention for infectious diseases such as COVID-19 [4].

However, the reliability of the information disseminated on social media can be occasionally questionable, leading to the generation and spread of misinformation [5]. The proliferation of health-related misinformation on social media poses a significant threat to public health and government stability and circulates rapidly across various platforms [2]. Such unreliable information can have long-lasting negative effects on the lives of people. Particularly in the healthcare domain, this may lead to serious damage. So accurate detection is important but, retrieving reliable and trustworthy information from the web takes time and effort and acts as an essential first stage in monitoring public health online [6]. Therefore, it is crucial to determine the accuracy of the information shared about COVID-19 to warn media users to suspect content [7]. Retrieving information from document collections containing misinformation is a major and big challenge. Users may need to help differentiate between accurate and inaccurate health information when using social media. The presence of misinformation may lead users to make poor decisions about their health situation. The TREC Health Misinformation track encourages research on information retrieval techniques that favor accurate and reliable information [8].

The most effective strategy for preventing the spread of misinformation is to fact-check the claims with reliable information from credible sources. Thus, false or misinformation, credibility, and fact-checking are interrelated terms [2]. Fact-checking is the process of evaluating the accuracy of information to determine its truthfulness and involves examining the facts to verify the legitimacy of the given information (9–10). Fact-checking can be achieved through automated text classification approaches which can be broadly categorized into rule-based, data-driven-based (Machine Learning/Deep Learning-based approaches), and hybrid approaches [11–13]. Automated fact-checking not only addresses the challenge of news integrity verification but also, helps to combat the spread of misinformation in today’s fast-paced media landscape. Thus, this approach has received considerable attention due to the proliferation of unreliable information (e.g., fake News, misinformation) on social media [10, 14]. Machine learning-based approaches have been significantly effective tools for distinguishing reliable information from misinformation in recent years [15]. These algorithms can verify the truth of data by comparing it to previously confirmed facts, and then categorize it as legitimate or illegitimate [16].

Despite the advantages of these approaches in detecting misinformation, they can lead to less accuracy or bias when handling sparse data, null values, or term frequencies. Addressing these challenges remains an active area of research [16]. The results of recent systematic reviews analyzing COVID-19 and other health-related datasets revealed differences in lexical and affective features [16, 17]. These analyses identified challenges in automatically detecting health-related misinformation and provided recommendations for future research. Studies highlighted the growth of content in social media during the pandemic, emphasizing the need for improved natural language understanding and text classification, especially in non-English languages [16]. Convolutional Neural Networks (CNN) based models, a type of deep learning architecture, have emerged as a powerful tools for text classification, particularly for short texts like tweets. Their ability to automatically identify patterns and key phrases in text makes them well-suited for this task [4, 18].

Previous studies have indicated that Twitter/X is a relatively fair social media platform with more than 199 million daily active users who can post, retweet, like, and comment within 280 characters, including links, videos, or images. Most of the messages are publicly available [19]. However, the rapid dissemination of misinformation about COVID-19 through this platform has resulted in several adverse consequences, including increased vaccine hesitancy, inappropriate medication use, and decreased trust in public health institutions [20–24].

Such misinformation can have life-threatening consequences by discouraging essential preventative (25–26). Assessing the extent and influence of misinformation is important for policymakers and public health organizations to predict population health behaviors. Therefore, in this study, we used Twitter/X to evaluate and compare different fact-checking models. This social media presents unique constraints due to the high volume, real-time nature, and inherent ambiguity of health information disseminated. We aimed to identify a more accurate and efficient approach to detecting health-related misinformation on this social media by performing a comparative analysis of the performance of these models. To achieve this, we collected, processed, labeled, and analyzed COVID-19 tweets collected from Twitter/X using relevant keywords and hashtags. The tweets were then classified into two distinct datasets: “Trustworthy information”, and “Misinformation”, through a labeling process. We employed the cosine similarity metric to address oversampling the minority class about tweets labeled as “Trustworthy information”, ensuring a more balanced representation of both trustworthy information and misinformation for training and testing purposes.

Finally, we analyzed and compared the performance of the various fact-checking models using standard performance metrics. These metrics included accuracy, precision, recall, and F1-score ROC curve, and AUC. By evaluating these metrics, we aimed to identify the model that demonstrated the most effective performance in detecting health-related misinformation about COVID-19 within the several proposed models.

The main Contributions of this study were:

1. Identifying the most effective approach for classifying reliable information versus misinformation health content shared on Twitter/X related to COVID-19.
2. Comparison of performance across different machine learning and deep learning models for evaluating fact-checking models.
3. Implementation of a novel approach to handle imbalanced datasets using cosine similarity.
4. Validation of the TextConvoNet model as the most effective for misinformation detection.

This paper is organized as follows:

Section II reviews previous studies related to detecting and fact-cheking health-related misinformation on social media and CNN-based models for text classification. Section III describes study method, the experimental setup and the fact-checking models used. Section IV discusses the results obtained. Section V is Discussion, Limitations, Implications of Researchand Future Works. Finally, Section VI provides the research conclusion.

## Literature review

Despite recent progress in misinformation detection methods, it seems that further investigation is required to develop more robust fact-checking models. Barve and Saini (2021), developed a healthcare misinformation detection model using machine learning classifiers like Naïve Bayes, which outperformed others in accuracy by analyzing sentimental and grammatical features [2]. Zeng et al. (2021), reviewed automated fact-checking and identified challenges such as narrow domains and imbalanced datasets [10]. Schlicht et al. (2024), highlighted the focus on COVID-19 misinformation detection, with limited studies on other health topics [13]. Anusree et al. (2022), introduced a social media fact-checking model, while El Kah and Zeroual (2023), reviewed Arabic COVID-19 datasets, guiding researchers toward trustworthy resources [14, 16]. Ni et al. (2023), found many health misinformation datasets emerging since 2020, especially for COVID-19, though definitions of misinformation remain unclear [27]. Khemani et al. (2024), showed superior performance of Graph Convolutional Networks (GCNs) in detecting misinformation [28]. Hangloo and Arora (2021), emphasized the role of CNN and RNN models in multimodal misinformation detection, while Comito et al. (2024), reviewed deep learning methods and called for addressing issues like explainability and cross-domain detection (29–30). Sikosana et al. (2024), evaluated machine learning (ML) and deep learning (DL) models for classifying COVID-19 misinformation on social media platforms, including Twitter/X [31]. Their findings indicated that advanced neural network approaches surpass traditional ML algorithms in detecting health-related misinformation. The study emphasized the need for optimized models capable of adapting to evolving misinformation narratives on social media platforms. Hussna et al. (2024), revealed that approximately 80% of studies on fake news detection related to COVID-19 on Twitter employed Deep Neural Networks [32]. While these networks enhance performance, they face challenges such as overfitting and higher prediction times. The study highlighted the necessity for large, robust training datasets and deeper community investigations to improve the classification and fact-checking of health-related information on social media. Chen et al. (2023), examined the adaptability and effectiveness of various deep learning models, including Long Short-Term Memory (LSTM), Bi-directional LSTM (Bi-LSTM), and Gated Recurrent Unit (GRU), across different text lengths and languages [33]. Their models achieved higher performance for English text compared to Chinese, underscoring the importance of linguistic adaptability in misinformation detection. Roy et al. (2023), developed an automated model using LSTM networks, integrating word embeddings such as CountVectorizer and TF-IDF [34]. Their model achieved an impressive accuracy of 99.82%, surpassing traditional ML models and existing DL approaches, thus demonstrating the potential of LSTM networks in capturing the nuances of misinformation in textual content. Conversely, Akhter et al. (2024), employed a CNN-based DL model for detecting COVID-19 fake news, achieving significant metrics such as a mean accuracy of 96.19%, a mean F1-score of 95%, and a high AUC-ROC of 98.5% [35]. These results illustrate the CNN model’s capability in handling the complexity of fake news content.

While the aforementioned studies demonstrate the efficacy of their respective models, a critical limitation lies in their applicability to real-world scenarios. Misinformation often involves evolving narratives and diverse formats, which present challenges for maintaining model accuracy in dynamic and heterogeneous online environments. Although achieving high accuracy, these models could benefit from broader evaluations of their performance in such scenarios.

This study seeks to address the gaps identified in prior research by uniquely integrating cosine similarity for data augmentation. This approach facilitates balanced datasets for trustworthy and misinformation classes, thereby enhancing classification performance. Furthermore, the proposed TextConvoNet model, with its parallel convolutional pathways, demonstrates superior performance compared to existing machine learning and deep learning techniques, offering a novel solution to the challenges of misinformation detection in complex, real-world contexts.

## Methods

### Study design and setting

This study aimed to identify the most effective approach for detecting and classifying reliable information versus misinformation in health content shared on Twitter/X, related to COVID-19 from 1 Jan 2020 to 30 June 2022. We used seven different machine learning/deep learning models. Tweets were collected, processed, labeled, and analyzed using relevant keywords and hashtags. Then classified into two distinct datasets: “Trustworthy information” versus “Misinformation”, through a labeling process. The cosine similarity metric was employed to address oversampling the minority of the trustworthy information class, ensuring a more balanced representation of both classes for training and testing purposes. Finally, the performance of the various fact-checking models was analyzed and compared using accuracy, precision, recall, F1-score, the ROC curve, and AUC.

### Data gathering

Firstly, we checked the literature and extracted a hashtag list about COVID-19. For data gathering, we used Lopez & Gallemore (2021) dataset, publicly available on Git Hub (https://github.com/lopezbec/COVID19_Tweets_Dataset) [29]. This dataset contains 2020–2022 COVID-19-related tweets published on Twitter/X. We have collected 11,896,788 tweets text using Twitter API software (https://developer.twitter.com/docs/twitter-api), from this data set with the below criteria:

English language tweets containing the following hashtags and with more than 5 likes and retweets were considered for review.

Hashtag lists: 2019_ncov, 2019ncov, corona, coronavirus, ncov2019, ncov_2019, coronaviruses, coronavirus_outbreak, coronavirus outbreak, coronavirus_updates, coronavirus updates, covid_19, covid19, ncov19, wuhan_virus, wuhan-virus, wuhanvirus, omicron_variant, omicronvariant.

For a comprehensive performance evaluation of various fact-checking approaches for text clustering, we used seven different machine learning/Deep learning techniques namely, Decision Tree (DT), Random Forest (RF), Support Vector Machine (SVM), Gated Recurrent Unit (GRU), Long Short-term Memory (LSTM), TextConvoNet, and a stacking ensemble learning with DT, RF, and SVM. Using these techniques helps establish the usability of the better model and increases the generalization of the results.

The stacking ensemble learning paradigm, such as DT, RF, and SVM as basic clustering models, can improve prediction accuracy [30, 36]. Previous research depicted that this approach leverages the complementary strengths of different models to construct a metamodel capable of capturing complex patterns in data [37].

### Implementation environment

All the experiments to examine the models’ performance are carried out on a system with a Dual-Core Intel Core i7 processor, 12 GB RAM, running Windows 10 operating system, with a 64-bit processor and NVidia K80 GPU kernel. All experiments were performed in the Google Colab environment with the use of Keras and Scikit Learn from Python 3.0 V.

### Used datasets

To conduct training and testing we used 7 various datasets containing labeled short texts and publicly available. The details of the used datasets are given in Table 1. In these datasets, usually more than two labels are provided for the data. Notably, only English-language cases and labels with certainties such as True and False have been extracted and used from this dataset.

**Table 1:** Details of the used datasets

Datasets	Number of data	Labels provided in the source	The labels used in the model	#Trustworthy information label	#Misinformation label
**DATASET-1: FaCov **[38]	3088	True, False	True, False	72	3016
**DATASET-2: FakeCOVID **[39]	7621	Collections, Correct, Correct attribution, Explanatory, Fake, Fake news, False, False and misleading, Half true, Half truth in dispute, labeled satire, Misattributed, Miscaptioned, Misinformation / Conspiracy theory, Misleading, Misleading/false, Mixed, Mixture, Mostly false, Mostly true, News, No evidence, Not true, Pants on fire, Partially correct, Partially false, Partially true, Partly false, Partly true, Scam, Suspicions, True, True but, Two pinocchios, Unlikely, Unproven, Unverified	Correct, Mostly true, True, News, True but, Half truth, Half true, Fake, Fake news, False, False and misleading, Mostly false, Misinformation / Conspiracy theory, Misleading, Misleading/False, Not true, Scam	88	7149
**DATASET-3: Check-COVID **[40]	1504	Not enough info, Refute, Support	Refute, Support	506	504
**DATASET-4: Esoc-covid-19-misinformation-dataset **[41]	5952	Conspiracy, Fake remedy, False Reporting	Conspiracy, Fake remedy, False reporting	0	4112
**DATASET-5: WHO Myth Busters **[42]	30	True	True	29	0
**DATASET-6: healthfeedback.org **[43]	784	True	True	765	0
**DATASET-7: Lopez and Gallemore **[29]	13,150	-	True, False	13,080	70

### Data cleaning and preprocessing

Data preprocessing is performed firstly by removing irrelevant data such as, duplicates, converting text to lowercase, and eliminating punctuations, stop words, single characters, and numbers. We extracted features from original datasets including tweet ID, body text, and labels, then we used tokenization and vectorization techniques to transform the word sequence into numerical representations. Basic vectorization methods such as TF-IDF and bag of words operate based on the frequency of word occurrences. The problem with these methods for large corpora is the creation of a high-volume sparse matrix as the final vector matrix. When hardware resource constraints exist, these methods are not practical or usable [44]. Therefore, in such cases, the use of Word embedding methods such as Skip-gram with negative sampling and GloVe, which assign numerical representations to words, are recommended. These methods are effective in quantifying societal biases and stereotypes in texts by overcoming the limitations of frequency-based approaches [45]. For applying TextConvoNet to generate word vectors from sentences, we used GloVe a pre-trained word embedding model [46]. In GloVe vectorization each word is associated with a vector representation based on co-occurrence probabilities of word pairs. This vectorization approach computes word vectors by considering the likelihood of simultaneous occurrences of two words, ultimately deriving the top 300 words with the highest co-occurrence probabilities for each word [47]. TextConvoNet recommends utilizing a vector length of 300 for each word [15].

To optimize hardware resources in this study, the vectorization of each word has been performed using only the initial 100 words. As a result, each text is transformed into a 100*100 matrix, reflecting the consideration of the initial 100 words in each text and a vector length of 100 for each word, thus leading to the mapping of each word to a 100-dimensional vector. After preprocessing, the tweet topics were extracted from the texts and classified as trustworthy information or misinformation.

### Cluster analysis to handle imbalance dataset

Cluster analysis refers to the application of computational and statistical methods to classify data. The goal of this process is to classify data into different clusters and make the similarity of cluster data as big as possible. In text clustering, some characteristics of data, such as the distribution of words, are used to facilitate classification [48]. Conventional text clustering methods are usually classified into four groups namely feature selection and transformation methods, distance-based clustering algorithms, word and phrase-based clustering, probabilistic clustering and topic models [49].

Due to the scarcity of data labeled as “Trustworthy information” compared to the data labeled as “Misinformation,” and highly imbalanced dataset. To address the oversampling of the minority class and increase the number of tweets in this class, we have utilized cosine similarity distanced-based clustering to identify more accurate data and ensure a more balanced representation of both classes, injected them into existing data resources with “Trustworthy information” label.

The utilization of the cosine similarity metric in text clustering provides the advantage of reducing the emphasis on irrelevant words and shows robustness against noisy data. This method has been leveraged for initial classification, improving the identification of precise data, and their integration into current data resources. Studies have shown that cosine similarity is effective in comparing textual content [50]. For this purpose, we randomly selected 250 tweets from Dataset 7 by Lopez and Gallemore (2021) using the shuffle function in Python [29]. These tweets were labeled using FactCheck.org and reuters.com tools. Subsequently, 180 tweets were labeled as “Trustworthy information” and 70 were labeled as “Misinformation”.

Trustworthy information labels were selected from this data and compared with tweets from the initial 6 months of 2020 to identify more trustworthy samples. In this comparison, all tweets that had a cosine similarity of over 0.3 were added to the final dataset for training and testing the network. The appropriate threshold for computing cosine similarity (0.3) was determined using trial and error. To do this, several thresholds (from 0.1 to 0.4 respectively) were measured and 2 medical librarians and information specialists familiar with fact-checking compared the validity of the results. These thresholds are reported in Table 2.

**Table 2:** Comparison of true positive and false positive in studied thresholds for “Trustworthy information” class

Threshold	0.1	0.15	0.2	0.25	0.3	0.35	0.4
True Negative	173	167	145	98	54	27	11
False Positive	66	61	48	33	18	9	6
**Precision**	**0.67**	**0.73**	**0.75**	**0.74**	**0.75**	**0.75**	**0.64**

The initial step involved the computation of the cosine similarity between each tweet and the entirety of the tweet corpus. Subsequently, the data was partitioned into two distinct clusters: one comprising tweets with similarity values less than the designated threshold, and the other encompassing those with similarity greater than the threshold. The target threshold was selected based on the precision parameter, which serves as a proxy for the classification accuracy of the trustworthy information class. To this end, the number of True Negatives (TN) and False Positives (FP) were tallied following each clustering iteration, enabling the calculation of the precision metric for each threshold considered.

A comparative analysis of the True Positive (TP) and False Positive (FP) rates was conducted across the various thresholds evaluated. At the 0.2 threshold, the data exhibited a heightened propensity for the injection of misinformation-labeled instances into the trustworthy information class, as evidenced by the elevated FP rate. Conversely, thresholds exceeding 0.4 were found to yield a negligible number of highly similar tweet clusters, rendering them unsuitable for practical application.

The 0.3 and 0.35 thresholds evaluation revealed no substantial difference in the percentage of positive and negative data injection. Importantly, the results were simultaneously reviewed by two medical librarians and information science specialists. The independent evaluations corroborated the findings and provided additional insights to inform the final threshold selection.

In regards to these findings, with 0.75 precision, 54 TP & 18 FP a threshold of 0.3 was ultimately chosen as the optimal parameter for identifying trustworthy information in our dataset. This decision was predicated on the need to strike a balance between minimizing the risk of contaminating the trustworthy information class and maximizing the injection of reliable data samples to enhance the training dataset.

After enhancing tweets in the “Trustworthy information” cluster, we tried to perform fact-cheking of main tweets with cosine similarity, so we categorized the main dataset into two clusters (Trustworthy information VS. Misinformation) with binary class text. then fact-cheking was performed.

### Implementation of the model

Of 7 experiment models, each model is characterized by specific hyperparameters. In machine learning, adjusting hyperparameters specific to the model is crucial for optimal performance, because these hyperparameters play a crucial role in controlling the complexity and overfitting of the models. For instance, in Convolutional Neural Networks (CNN), these hyperparameters include the number and type of layers, the number of convolutional operators (filter size), dimensions of convolution kernels (kernel size), training epochs, batch size, and other related factors, but in Decision tree-based models, hyperparameters include maximum tree depth and the number of trees used. In the context of this study, default parameters have been employed for DT, RF, SVM, and ensemble learning models. GRU and LSTM models were implemented using one and two-layer recurrent networks with varying numbers of cells. (16, 32, 64, and 128). To prevent overfitting, common methods like Dropout and regularization techniques such as L1 and L2 were employed, but it was not successful and overfitting occurred [51].

### TextConvoNet model

**Fig. 1:**
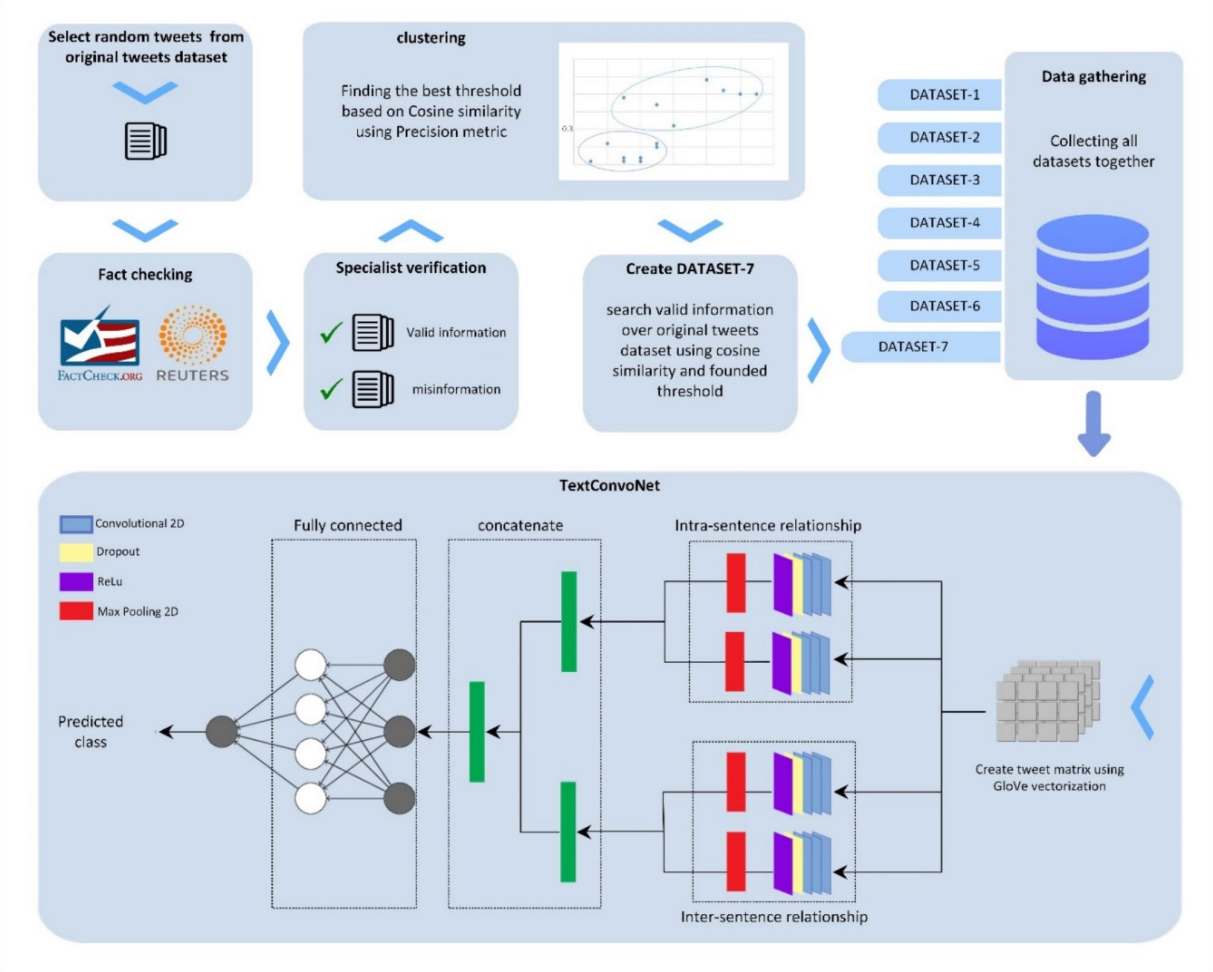
The schematic model of the study process and TextConv model architecture

Convolutional neural networks are one of the text classification methods that are very effectively used to solve the problem of text classification [52]. The result of the classification is the distribution of the probabilities that the text belongs to be forehand defined classes. We used the TextConvoNet model that was proposed by Soni et al.(2023) [15],. Their proposed architecture uses a 2-dimensional convolutional filter to extract the intra-sentence and inter-sentence n-gram features from text data. First, it represents the text data as a paragraph-level (multi-sentence) embedding matrix, which helps in applying 2-dimensional convolutional filters. Thereafter, multiple convolutional filters are applied to the extracted features. The resultant features are concatenated and fed into the classification layer for classification purposes. TextConvoNet architecture, illustrated in Fig. 1, includes four parallel convolutional subnetworks known as pathways. Each pathway contains three 2-dimensional convolutional layers, one Relu layer, and a 2-dimensional Max pooling layer.

The first two pathways, which conduct data as a main pathway, are concatenated and utilized to obtain an intra-sentence relationship. Meanwhile, the second two pathways, also known as the second main pathway, are concatenated to obtain an inter-sentence relationship. Finally, these two main pathways are concatenated, and the classification is carried out in a fully connected layer. TextConvoNet did not suggest utilizing dropout. Dropout means temporarily leaving out some neural network neurons from calculations in each iteration of the training phase. These neurons are randomly selected and removed from the network architecture. This method has been very successful in preventing overfitting. Since some neurons are removed from the process of training and calculations of network weights, excessive training will not happen on these neurons, and eventually overfitting is avoided [53]. However, in this study dropout was used before the Relu layer in all pathways to prevent overfitting. The dropout rate of 0.5 was selected in this research. The model was trained on a batch size of 128 with a learning rate of 0.0001. Adam optimizer was used with the Binary Cross-Entropy loss function (BCE). Model hyperparameters and settings are presented in Table 3.

**Table 3:** TextConvoNet model hyperparameters values

Hyperparameters			
Main pathway	Subdivided pathway		size kernel
Inter-sentence	1		2*1
	2		2*2
Intra-sentence	1		1*2
	2		1*3
**Setting**			
Input arrays’ size		100*100	
Number of fully connected network layer neurons		128	
Loss function		Binary cross entropy	
Learning rate		0.0001	
Optimizer		Adam	
Batch size		128	

### Performance evaluation

In the experimental evaluation, we used 4 standard measures due to their broad applicability, to evaluate the performance of the prediction models. Performance measures were accuracy, precision, recall, and f-score. A detailed description of these performance measures is presented in Table 4. As one of the most widely applied and applicable tools for model evaluation and selection, The k-fold cross-validation method was used to enhance the assessment of results TextConvoNet model.

**Table 4:** Description of the performance evaluation metrics

Metric	Formulation	Defination
Accuracy	$\:\frac{TP+TN}{TP+TN+FP+FN}$	Accuracy refered to the amount of accurate assumptions the algorithm produced for forecasts of all sorts.
Precision	$\:\frac{TP}{TP+FP}$	Precision was the percentage of successful cases that were reported correctly.
Recall	$\:\frac{TP}{TP+FN}$	It was the number of right positive outcomes divided by the number of all related samples (including samples that were meant to be positive).
F 1-score	$\:2\text{*}\frac{\left(\text{P}\text{r}\text{e}\text{c}\text{i}\text{s}\text{o}\text{n}\text{*}\text{R}\text{e}\text{c}\text{a}\text{l}\text{l}\right)}{\left(\text{P}\text{r}\text{e}\text{c}\text{i}\text{s}\text{o}\text{n}+\text{R}\text{e}\text{c}\text{a}\text{l}\text{l}\right)}=\:\frac{TP}{TP+\:\frac{1}{2}(FP+FN)}$	It was the harmonic mean of the precision and recall values.

TN = True Negatives

TP = True Positives

FP = False Positives

FN = False Negatives

## Results

The results of the study demonstrated that among the text classifier used models, the TextConvoNet model produced significant result values and exhibited the best performance among the models for health misinformation detection tasks on Twitter/X. For measures of accuracy, precision, F1 score, and recall the average values of TextConvoNet were 90.28, 90.28, 90.29, and 0.9030, respectively. The ROC AUC score was 0.901. These values were higher than those obtained by other machine learning and deep learning-based models that were investigated, indicating robust discriminatory power. Table 5 presents the findings of the machine learning and deep learning models evaluated in terms of their performance metrics.

**Table 5:** Classification results of the baseline models (TextConvoNet, DT, RF, SVM, and stacking ensemble of DT, RF, SVM) for different performance measures

Models	Accuracy	F Score	Precision	Recall	ROC AUC
TextConvoNet	**90.28**	**90.29**	**90.28**	**90.30**	0.901
DT	74.72	73.62	72.94	74.33	0.749
RF	80.16	78.15	81.90	74.73	0.874
SVM	69.15	67.50	67.53	67.47	0.687
Stacking ensemble (DT, RF, SVM)	80.42	78.93	80.70	77.23	0.864
GRU*	Overfitted				
LSTM*	Overfitted				

_*GRU and LSTM were over−fitted due to training data rapidly_

The results of the 5-fold cross-validation, presented in Table 6, further reinforced the robustness of the TextConvoNet architecture, with an accuracy of 89.03%, precision of 89.06%, recall of 88.94%, and F1 score of 89.0%.

**Table 6:** The k-fold cross-validation results in the TextConvoNet model

Cross-validation method	Accuracy	F Score	Precision	Recall
5-fold	89.03	89.0	89.06	88.94

**Fig. 2:**
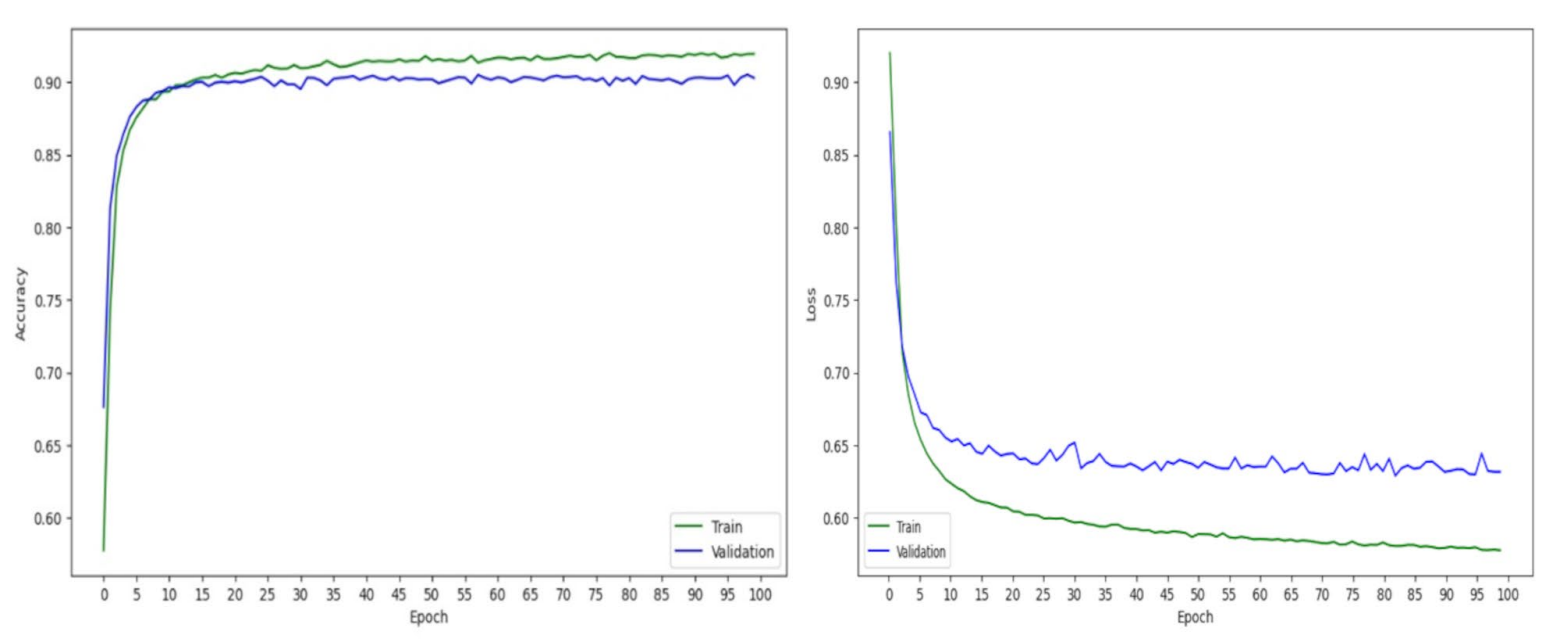
Loss and accuracy graph of TextConvoNet model on training and validation

Fig. 2 illustrates the evolution of TextConvoNet’s loss and accuracy throughout the training process. The loss value was reduced and the model did not overfit. The accuracy curve indicated a consistent increase in accuracy over successive epochs, with a plateau occurring around epoch 5. This outcome aligns with the decision to terminate the training process after five epochs to reduce the risk of overfitting, as evidenced by the model’s superior performance on the held-out validation set.

To gain a more profound understanding of the model’s effectiveness, we conducted a detailed analysis of the results in Table 7. The “Trustworthy information” class achieved an accuracy of 85%, precision of 93%, recall of 86%, and F1 score of 89% for the TextConvoNet algorithm. Moreover, its performance in the Misinformation category was even more impressive, with an accuracy of 94%, precision of 88%, recall of 94%, and F1 score of 91%.

**Table 7:** The performance comparison between the baseline models on trustworthy information vs. misinformation classes

Models	Class	Accuracy		F Score	Precision	Recall
TextConvoNet	Trustworthy information		0.85	0.89	0.93	0.86
	Misinformation		0.94	0.91	0.88	0.94
DT	Trustworthy information		0.74	0.74	0.73	0.75
	Misinformation		0.75	0.76	0.77	0.75
RF	Trustworthy information		0.74	0.78	0.82	0.75
	Misinformation		0.84	0.82	0.79	0.85
SVM	Trustworthy information		0.67	0.67	0.68	0.67
	Misinformation		0.70	0.71	0.71	0.71
Stacking ensemble (DT, RF, & SVM)	Trustworthy information		0.77	0.79	0.81	0.78
	Misinformation		0.83	0.82	0.81	0.83

**Fig. 3:**
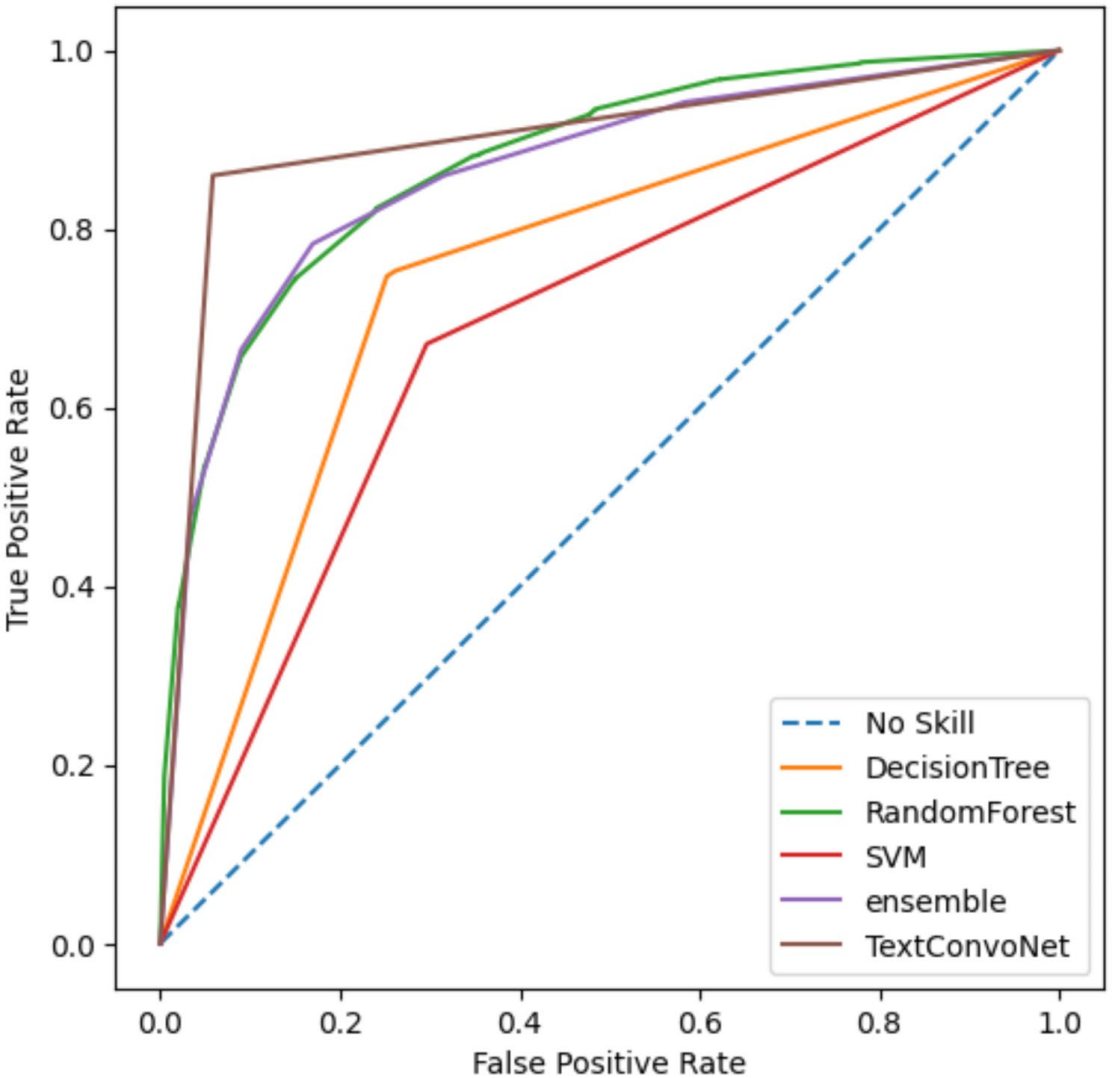
ROC curve of baseline models

Fig. 3 presents the receiver operating characteristic (ROC) curve for the studied models, allowing for a comparative analysis of the false positive and true positive rates. The area under the ROC curve for the DT, SVM, RF, Stacking ensemble, and TextConvoNet models is 0.749, 0.687, 0.874, 0.869, and 0.901, respectively. AUC (Area Under the Curve) could be used to effectively compare the performance of methods in binary classification. Considering the results from Table 5; Fig. 3, the TextConvoNet method was effective in classifying the data compared to other methods.

## Discussion

In this study, we employed a range of text classifier models, including DT, RF, SVM, GRU, LSTM, TextConvoNet, and a stacking ensemble learning approach integrating DT, RF, and SVM, to classify health information related to COVID-19. Our goal was to identify the most effective approach for detecting and classifying trustworthy information V.S misinformation health content shared on Twitter/X related to COVID-19. This research contributes to the growing body of work on combating health misinformation and offers insights into improving automated fact-checking approaches. The results of the study demonstrated that TextConvoNet produced significant result values and exhibited the best performance among the models for health misinformation detection tasks on Twitter/X. Specifically, TextConvoNet achieved superior performance metrics, such as an accuracy of 90.28% and an ROC AUC score of 0.901, which reflect its robust discriminatory power. These results are along with Soni et al. (2023) [15],. They compared TextConvoNet with other machine learning, deep learning, and attention-based models. Their results declared that the presented TextConvoNet outperformed and yielded better performance than the other used models for text classification purposes.

The effectiveness of TextConvoNet can be attributed to its unique architectural design, which leverages parallel convolutional pathways to capture both intra-sentence and inter-sentence relationships. The 2D convolutional filters efficiently extracted localized n-gram features and long-range semantic dependencies, making the model adept at distinguishing reliable health information from misinformation in the noisy and concise language of tweets. This is consistent with other studies that have highlighted the efficacy of CNN models for short-text classification tasks tasks such as sentence classification, particularly in social media contexts [52, 56–59].

Social media texts often contain noise, such as creative and novel phrases, sarcastic emoji expressions, and misspellings. Additionally, the class imbalance issue is a serious problem. To address these challenges, Luo et al. (2022), constructed a COVID-19 personal health mentions (PHM) dataset comprising over 11,000 annotated tweets as a text classification task, and proposed a dual convolutional neural network (CNN) structure to address the concerns [4]. The dual CNN effectively utilized the auxiliary information extracted by the A-Net to address the class imbalance problem in the dataset. The effectiveness of the dual CNN in identifying PHMs, particularly those crucial for public health surveillance, was observed.

Scott and Matwin (1999), examined some alternative ways to represent text based on syntactic and semantic relationships between words [11]. Their results showed that advancement in this field lies in the development of innovative learning algorithms and techniques for integrating existing learners. More advanced Natural Language Processing techniques could generate better text representations.

According to our results, TextConvoNet was able to identify discriminative patterns to differentiate reliable health-related information from misinformation. Furthermore, there was a discernible inclination towards more accurate identification of misinformation content.

In this study, ROC analysis revealed that the model showed an impressive capacity for accurately differentiating between trustworthy information and misinformation disseminated via Twitter/X. These findings are consistent with previous studies that have demonstrated the efficacy of CNN models for text classification, particularly when applied to short-form social media data [4, 18].

In contrast, LSTM and GRU models faced overfitting despite the use of dropout and regularization techniques, as observed in this study. This outcome highlights the challenges of training deep neural networks on imbalanced and noisy datasets. Other research declared that both LSTM and GRU models could face overfitting, a common problem in machine learning that can lead to inaccurate predictions and generalization. Researchers have addressed this problem by using methods such as dropout to reduce overfitting by weakening the connections between neurons (54–55, 60). This finding is in line with previous research indicating that deep neural networks may be less adept at generalization on highly skewed and noisy text data [61–63]. This indicates the necessity for the implementation of additional regularization techniques, data augmentation, or class-balanced sampling methods that are specifically designed for the detection of health misinformation [64–66].

Furthermore, despite the achievements of the stacking model, its effectiveness is highly dependent on the diversity and quality of its base learners. This highlights the critical role of careful model selection and optimization.

It has been demonstrated that the use of text similarity methods such as Cosine similarity for data augmentation, is a valuable approach for addressing class imbalance and enriching the “Trustworthy information” class. This approach involves clustering tweets based on their textual similarity with more diverse and representative samples can lead to a more robust training dataset and improve the model’s ability to cluster [50].A significant challenge identified in this study was the class imbalance issue, which hindered the performance of certain models.To address this, future research could explore advanced data augmentation strategies, such as Cosine similarity-based clustering and expert-guided threshold tuning, which have proven effective in enriching training datasets [67–69].

### Limitations

This study was faced with several limitations as below:

1. **Data Collection**: Internet restrictions and Twitter/X filtering in Iran significantly delayed data collection.
2. **Embedding Models**: The lack of domain-specific pre-trained GloVe embeddings may have affected semantic understanding.
3. **Generalizability**: While the model performed well on English tweets, its effectiveness in other languages remains to be tested.

### Implications of research

The findings of this study have significant implications for advancing public health initiatives and misinformation management. The TextConvoNet model’s high accuracy and robust performance can empower researchers to develop scalable systems for real-time misinformation detection. Public health agencies could utilize such models for timely intervention, enhancing trust and compliance during health crises.

### Future work

Future studies could explore the use of more advanced contextual language models, such as BERT, to enhance detection capabilities. In addition, while the multi-dataset approach provides a strong foundation for model training and can result in robust training datasets, future studies could investigate its applicability in different health misinformation domains. The application of contextual embedding methods for improved semantic understanding, as well as the feasibility of integrating cross-lingual datasets to expand the model’s utility, can also be considered areas for future research.

## Conclusion

This study has identified the most effective approach for classifying reliable information versus misinformation shared on Twitter/X related to COVID-19. By conducting a comparative analysis of multiple machine learning and deep learning models, this research highlights the superior performance of the TextConvoNet model in misinformation detection. The implementation of a novel approach to address dataset imbalances using cosine similarity further strengthens the robustness of the proposed methodology.

We believe that this study can make an important contribution to reducing the public health risk of widespread health misinformation on social media. The high accuracy of the TextConvoNet model in automatically verifying health information from tweets can facilitate real-time monitoring and timely intervention by health regulators and policymakers.

Consequently, these findings offer actionable insights for researchers and public health agencies, enabling the development of real-time misinformation detection systems that can be utilized during public health crises.

## Data Availability

For data gathering, we used a dataset publicly available on Git Hub at: (https://github.com/lopezbec/COVID19_Tweets_Dataset). So, informed consent was not required for this study. The data that support the findings of this study are available from the corresponding author upon reasonable request.

## Abbreviations

DT: Decision Tree
RF: Random Forest
SVM: Support Vector Machine
GRU: Gated Recurrent Unit
LSTM: Long short-term memory
TN: True Negatives
FP: False Positives
TP: True Positive
FN: False Negatives
CNN: Convolutional Neural Networks
BCE: Binary Cross-Entropy loss function
ROC: Receiver Operating Characteristic
AUC: Area Under the Curve
